# Verbal fluency tasks and attention problems in children with ADHD: evidence from fNIRS

**DOI:** 10.3389/fneur.2025.1541589

**Published:** 2025-07-02

**Authors:** Zouji Bian, Xiquan Ma, Yanhan Wang, Xiaodan Yu

**Affiliations:** Department of Developmental and Behavioral Pediatrics, Shanghai Children’s Medical Center, School of Medicine, Shanghai Jiaotong University, Shanghai, China

**Keywords:** ADHD, fNIRS, VFT, attention, children

## Abstract

**Background:**

Functional near-infrared spectroscopy (fNIRS) is a promising non-invasive neuroimaging tool for assessing cortical activity in children with attention-deficit hyperactivity disorder (ADHD). While Verbal Fluency Tasks (VFTs) are widely used in adolescents and adults, their application in younger children remains underexplored. This study aimed to examine cortical activation during a VFT in children with ADHD and its association with attention-related behavioral symptoms.

**Methods:**

Thirteen children with ADHD (aged 7–9) and 13 matched neurotypically developed controls completed a VFT while undergoing fNIRS. Activation in the dorsolateral prefrontal cortex (DLPFC) was analyzed using mean amplitude, center of gravity (COG), and initial slope. Associations with the Swanson, Nolan, and Pelham Rating Scale (SNAP-IV) and Diagnostic and Statistical Manual of Mental Disorders, Fifth Edition (DSM-V) scores were examined.

**Results:**

Children with ADHD showed significantly lower DLPFC activation and widespread negative patterns compared to neurotypically developed peers (*p* < 0.05). Mean activation amplitude was significantly correlated with inattention scores on both the SNAP-IV and DSM-V scales.

**Discussion:**

fNIRS revealed altered DLPFC activation in children with ADHD during VFT, underscoring its potential as an objective tool to support clinical assessment of attention deficits in younger populations.

## 1 Introduction

Attention-deficit hyperactivity disorder (ADHD) is a prevalent neurodevelopmental disorder characterized by persistent patterns of inattention, hyperactivity, and impulsivity that interfere with functioning or development. Children with ADHD exhibit deficits in executive functions, including impaired verbal fluency, working memory, and cognitive flexibility, which manifest as difficulties in academic performance and social interactions (1). Additionally, behavioral symptoms such as frequent task switching, lack of sustained attention, and excessive motor activity make clinical diagnosis and functional assessment particularly challenging (2). Despite advancements in diagnostic tools, the subjective nature of symptom evaluation—relying heavily on parent- and teacher-reported questionnaires—poses a significant limitation to accurately identifying ADHD-related deficits (3). This underscores the need for objective, neurophysiological measures that can complement traditional assessments, offering insights into the underlying mechanisms of cognitive dysfunction in children with ADHD.

Neuroimaging techniques have been extensively employed to explore the neural correlations of ADHD. Functional imaging methods, such as functional magnetic resonance imaging (fMRI) and electroencephalography (EEG) (4), have revealed hypoactivation in the prefrontal cortex (PFC) and default mode network (DMN) during tasks requiring sustained attention, alongside altered connectivity patterns between task-positive and task-negative networks (5). In contrast, functional near-infrared spectroscopy (fNIRS) measures changes in oxygenated and deoxygenated hemoglobin within the cerebral cortex, offering insights into brain activity with high temporal resolution (6). Compared to other neuroimaging techniques, fMRI or EEG, fNIRS provides a unique balance of portability, cost-effectiveness, and tolerance for movement artifacts, making it particularly well-suited for research involving children with neurodevelopmental disorders (7, 8). Furthermore, its non-invasive nature and use of near-infrared light minimize discomfort, increasing compliance among pediatric populations.

In the context of ADHD, fNIRS has been increasingly utilized to investigate cortical hemodynamic responses during cognitive tasks, such as verbal fluency or sustained attention tasks (9, 10). Studies highlight altered activation patterns in the PFC, a region critical for executive functioning, which aligns with the theoretical underpinnings of ADHD-related deficits. For instance, a study demonstrated reduced PFC activity in children with ADHD during working memory tasks, correlating with diminished task performance (9). Such findings suggest that fNIRS can serve as an objective biomarker to assess functional impairments in ADHD, offering a complementary perspective to traditional behavioral assessments.

Recently, fNIRS has emerged as a valuable tool that can be utilized to assess cortical activity in children with ADHD during cognitive tasks. Studies utilizing fNIRS consistently demonstrate diminished PFC activation during tasks involving verbal fluency, working memory, and inhibitory control, corroborating the executive dysfunctions observed in ADHD populations (11, 12). Reduced hemodynamic responses have been observed in the PFC of children with ADHD during a Go/NoGo task, highlighting deficits in inhibitory control—a hallmark of the disorder (13). Furthermore, fNIRS studies have indicated that these functional impairments are not static but may fluctuate with task complexity and external demands, underscoring the dynamic nature of neural activity in ADHD (14).

fNIRS, therefore, may significantly advance the assessment of children with ADHD. However, before applying it to the assessment of core symptoms, it is crucial to understand brain activation differences between ADHD and neurotypically developed children. To address the limitations of traditional assessments and leverage the advantages of fNIRS in pediatric populations, we selected the Verbal Fluency Task (VFT) as a cognitive paradigm well-suited for the evaluation of executive function in children. By examining cortical activation during VFT using fNIRS, this study aimed to uncover neural markers associated with attentional deficits in children with ADHD.

## 2 Materials and methods

### 2.1 Participants

From January to September 2022, 13 children with ADHD aged 7–9 years old and 13 age- and gender-matched neurotypically developed controls were recruited from the Shanghai Children’s Medical Centre. To ensure comparability between groups and reduce potential confounding effects, participants in the ADHD and neurotypically developed groups were individually matched on age (7–9 years), sex, and intellectual function. All participants completed the Chinese version of the Wechsler Intelligence Scale for Children (WISC), and only those with a full-scale IQ ≥ 70 were included. Group-level comparisons confirmed no significant differences in language IQ, performance IQ, or total IQ between the ADHD and neurotypically developed groups (all *p* > 0.05).

We included children in the ADHD group who were clinically diagnosed by licensed pediatric psychiatrists according to the DSM-V criteria, and symptom severity was further assessed using the Swanson, Nolan, and Pelham Rating Scale (SNAP-IV) and the Weiss Functional Impairment Rating Scale-Parent Form (WEISS-P). Neurotypically developed participants were recruited through school-based screening and confirmed to have no history of neurological, psychiatric, or developmental disorders. We excluded all children with (1) current or prior treatment with stimulant medication or behavioral therapy; (2) comorbid diagnoses such as autism spectrum disorder, specific learning disorder, or language impairment; (3) history of epilepsy, traumatic brain injury, or other major neurological or systemic conditions; (4) uncorrected vision or hearing impairments; (5) inability to complete the task due to comprehension or behavioral issues; and (6) poor fNIRS data quality resulting from excessive motion artifacts or channel loss. The detailed assessment information for both the ADHD and control groups is presented in Table 1. Written informed consent was obtained from all participants, and the study was approved by the Medical Ethics Committee of Shanghai Children’s Medical Center affiliated with Shanghai Jiao Tong University School of Medicine (SCMCIRB-Y2020136).

**Table 1:** Demographics of participants.

Patient characteristics	ADHD group (n = 13)	Control group (n = 13)	*p*
	Mean ± SD, %	Mean ± SD, %	
Sex (% male)^*^	0.69	0.85	0.64
Age (year)	9.00 ± 1.84	8.62 ± 1.78	0.62
WISC-R			
Language IQ	88.08 ± 15.05	98.15 ± 19.69	0.17
Operational IQ	95.38 ± 27.58	105.92 ± 15.39	0.26
Total IQ	93.23 ± 12.50	101.85 ± 18.11	0.19

*p < 0.05.

### 2.2 Experimental procedure

**Figure 1:**
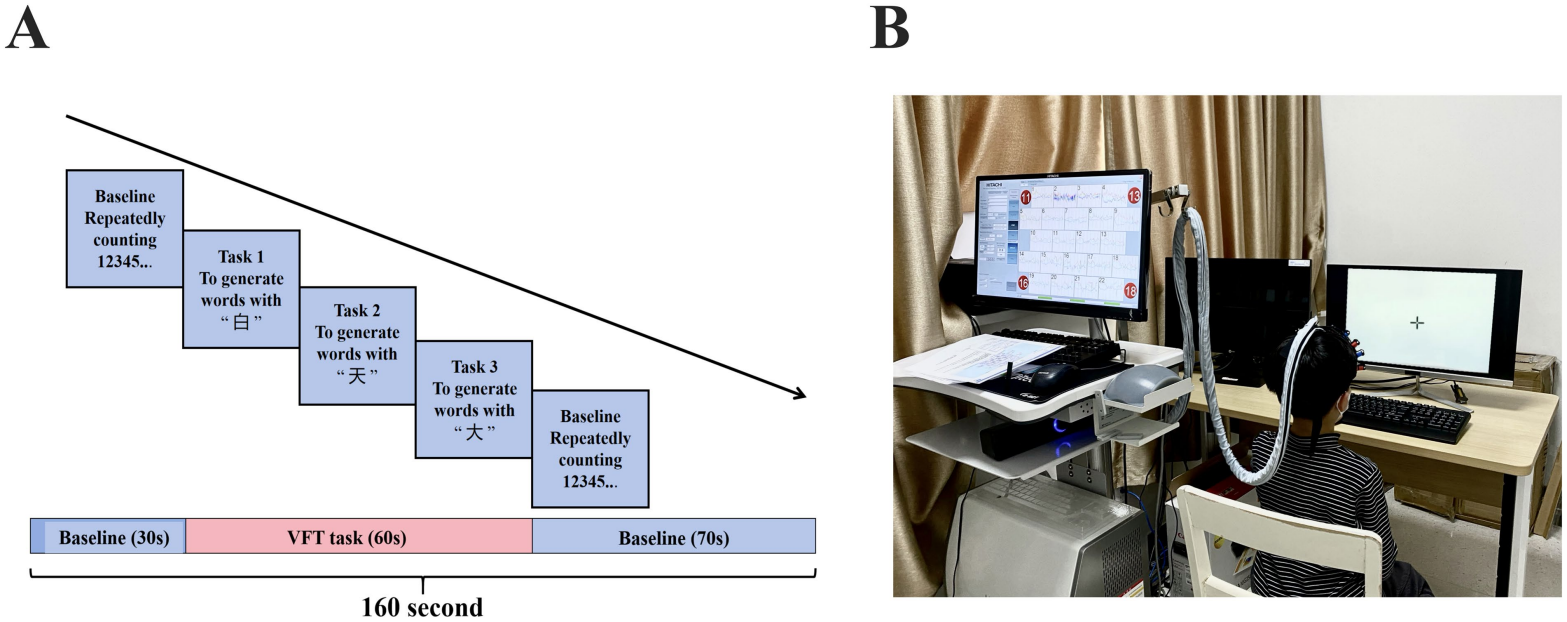
fNIRS tasks. **(A)** Verbal fluency test task. **(B)** Scanning setup.

During the fNIRS scanning, all participants completed a VFT task (Figure 1). The test was conducted in a quiet and comfortable environment. To minimize motion-related artifacts in participants, all fNIRS caps used in this study were child-sized and designed to fit the average head circumference of 8-year-old children. Optodes were securely affixed to the scalp using adjustable elastic straps and soft, foam padding to ensure stable contact and optimal light transmission throughout the recording. This setup helped reduce signal noise caused by movement and maintained data quality across participants.

Throughout the experiment, trained researchers remained present to supervise and support each participant during task execution. Prior to data acquisition, task instructions were explained clearly and a practice trial was provided to ensure comprehension. During the task, verbal prompts were given in a neutral tone when participants appeared distracted or paused speaking, to maintain engagement and optimize compliance without introducing bias. The VFT task consisted of three consecutive phases: a 30-s pre-task baseline, a 60-s task period, and a 70-s post-task baseline (Figure 1A). During the pre-task baseline, participants were instructed to repeatedly count from 1 to 5. During the task period, participants were asked to generate as many words as possible using the Chinese characters for “white,” “sky,” and “big.” To prevent extended pauses, the three given characters were changed every 20 s during the 60-s task period. Finally, in the post-task baseline, participants repeated the action of counting from 1 to 5. Participants’ verbal responses during the VFT were audio-recorded and transcribed offline (Figure 1B). The number of valid words generated in each 20-s sub-block was counted, and trials with fewer than five words across the entire task period were excluded from the fNIRS analysis to ensure sufficient task engagement. To control for potential order effects in cognitive performance, the sequence of task presentation was randomized across participants, reducing the influence of fatigue, learning effects, or anticipation bias on neural activation outcomes.

**Figure 2:**
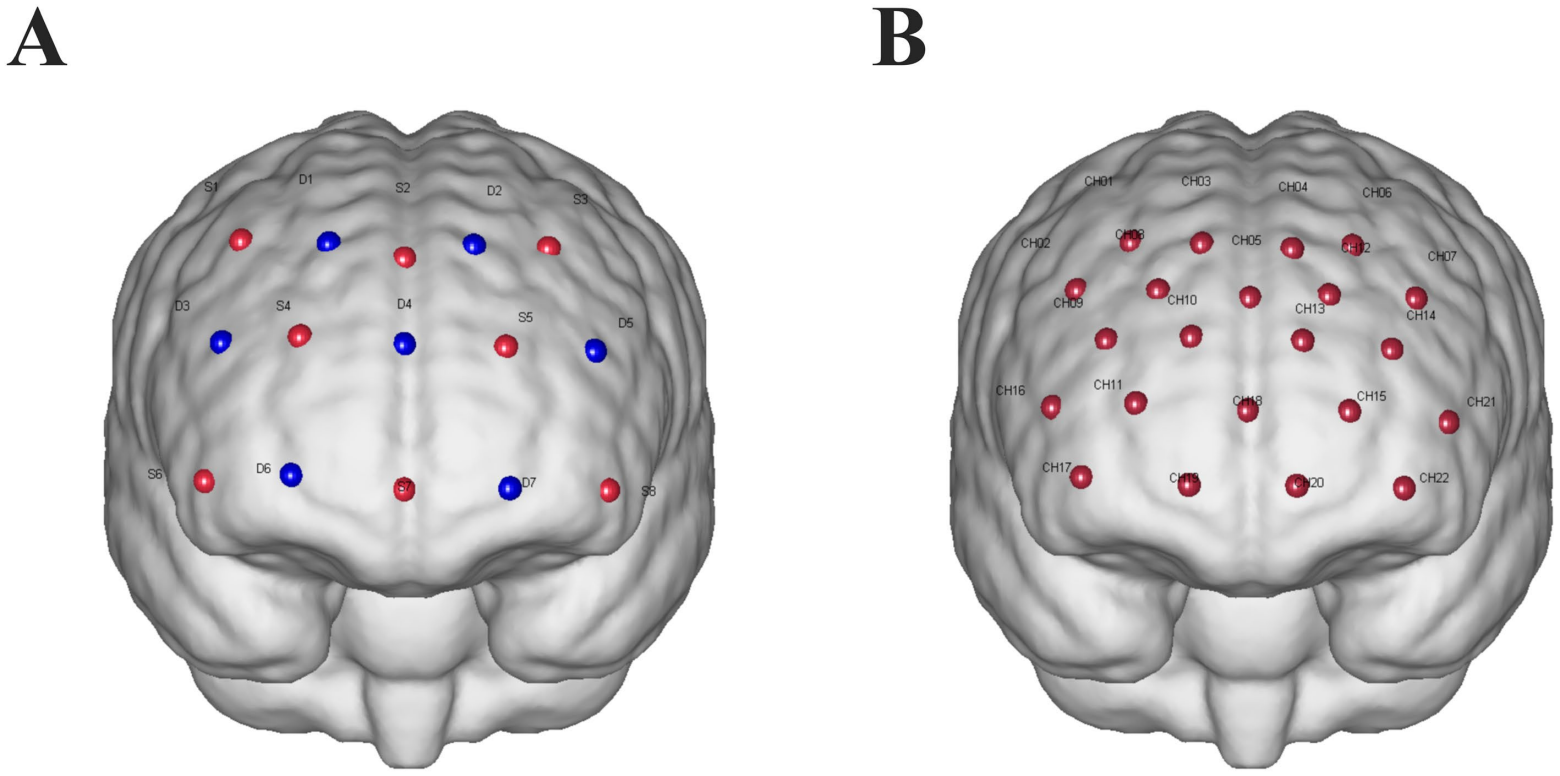
**(A)** fNIRS probe design: sources are represented by red dots labeled with “S” and a number, while detectors are represented by blue dots labeled with “D” and a number. The optodes are arranged with a 30-mm spacing between them. **(B)** Channels are indicated by red dots labeled “CH” followed by a number.

Continuous-wave fNIRS measurements (ETG-one, Hitachi, Ltd.) were used to capture cortical oxyhemoglobin (HbO) deoxyhemoglobin (HbR) signals from participants. The sampling rate was set to 11 Hz, with wavelengths of 730 nm and 850 nm. The cap used for brain imaging included a 22-channel probe (eight emitters and seven detectors) covering bilateral frontal areas. To obtain Montreal Neurological Institute (MNI) coordinates for each fNIRS channel, representing the midpoint positions of the source-detector pairs, an electromagnetic three-dimensional (3D) digitization system (FASTRK, Polhemus, Colchester, VT) was used to record spatial coordinates of the sources, detectors, and anchor points (Cz, Nz, Iz, AL, and AR) (Figure 2).

### 2.3 fNIRS data analysis and statistics

The fNIRS data were preprocessed and analyzed using the Homer2 software package (15). Raw light intensity signals recorded at two wavelengths (730 nm and 850 nm) were first converted into optical density. Subsequently, concentration changes in oxyhemoglobin and deoxyhemoglobin were calculated using the modified Beer–Lambert law, with a differential path length factor (DPF) of 6. Although DPF can vary by age, a value of 6 is widely used in pediatric studies (16, 17). To minimize noise and motion-related artifacts, a 0.01–0.2 Hz band-pass filter was applied to remove low-frequency drifts (e.g., Mayer waves, respiration) and high-frequency physiological noise (e.g., cardiac signals). Motion artifacts were identified both visually (sudden spikes or baseline shifts) and algorithmically, and were corrected using spline interpolation and wavelet-based filtering. Baseline correction was performed via linear detrending, using two stable reference periods: the last 10 s of the pre-task baseline and the 50–55-s interval after the task. Channels were excluded from further analysis if the HbO amplitude was below 0.01 μM, if the signal showed reversed polarity (e.g., downward response during the task), or if the coefficient of variation exceeded 15%. Only clean, reliable channels were retained for further activation extraction. The oxyhemoglobin signal was used to represent the results because it typically has a better signal-to-noise ratio than the deoxyhemoglobin signal (18).

Task-related cortical activation was quantified using three key temporal features extracted from the HbO signal: mean activation amplitude, center of gravity (COG), and initial slope. These metrics were chosen to capture the intensity and timing of the hemodynamic response. (1) Mean activation amplitude: this metric reflects the overall strength of the hemodynamic response during task execution, calculated as the average HbO concentration within the 60-s task period (from 30 to 90 s), after subtracting a linear baseline. The baseline was defined by fitting a linear trend using two reference segments: the final 10 s of the pre-task period (20–30 s) and the 50–55 s segment of the post-task period (90–145 s). The subtraction of this trend removes slow drifts and offsets in the signal. The resulting mean amplitude represents the net increase in cortical oxygenation during verbal generation. (2) COG: mean activation amplitude COG measures the temporal centroid of the positive HbO response across the full trial duration (0–125 s). COG is expressed in seconds and reflects the timing of peak engagement. A lower COG indicates early peak activation, while a higher COG suggests sustained or delayed activation. (3) Initial slope: to estimate the rapidity of the cortical response to task onset, the initial slope of the HbO curve was calculated over the first 5 s of the task period (30–35 s). A linear regression was fitted to the HbO signal over this window, and the slope coefficient was used as the feature (19). This parameter reflects how quickly prefrontal cortical regions begin to respond to the cognitive demand. Each of these features was calculated separately for the 22 channels. To enhance interpretability, channels were grouped into predefined left/right dorsolateral prefrontal cortex (DLPFC) based on Montreal Neurological Institute coordinate registration and 3D optode localization; feature values were obtained by averaging across valid channels. For group-level comparisons (ADHD vs. Neurotypically Developed), two-sample *t*-tests were used with false discovery rate (FDR) correction for multiple comparisons. In addition, Spearman correlation analyses were performed to examine the relationships between fNIRS activation metrics and behavioral symptom scores (SNAP-IV and DSM-V). Data analysis was performed using SPSS version 26.0 (IBM Corporation, Armonk, NY), with *p* < 0.05 indicating statistical significance. These methodological components enabled a detailed analysis of hemodynamic responses in prefrontal regions during a structured VFTs.

## 3 Results

### 3.1 Demographics and clinical characteristics

Based on the demographic data, the ADHD group consisted of 13 participants with a mean ± standard deviation age of 9.00 ± 1.84 years, while the control group included 13 participants with a mean age of 8.62 ± 1.78 years. Gender distribution was similar between the groups, with the ADHD group comprising nine males and four females, and the neurotypically developed group comprising 11 males and two females. There were no significant differences between the ADHD and control groups in terms of language IQ (ADHD: 88.08 ± 15.05; CG: 98.15 ± 19.69), operational IQ (ADHD: 95.38 ± 27.58; CG: 105.92 ± 15.39), and total IQ (ADHD: 93.23 ± 12.50; CG: 101.85 ± 18.11). All *p*-values for these comparisons were greater than 0.05, indicating no significant differences between the two groups in these baseline characteristics.

Scales used in this study included DSM-V, SNAP-IV, and WEISS (Table 2). Patients with ADHD showed impairment in at least one functional domain on these scales compared to neurotypically developed individuals. Significant differences were observed in SNAP-IV (*p* < 0.001), DSM-V (*p* < 0.01), and WEISS (*p* < 0.05).

**Table 2:** Clinical characteristics.

Scales	ADHD (*N* = 13)	Control (*N* = 13)	*p*
DSM-V (inattention)	7.15 ± 1.03	2.77 ± 1.25	0.000***
DSM-V (hyperactivity)	4.77 ± 1.93	2.00 ± 1.25	0.002**
SNAP-IV (inattention)	2.09 ± 0.47	0.93 ± 0.44	0.000***
SNAP-IV (hyperactivity)	1.80 ± 0.45	0.76 ± 0.73	0.000***
SNAP-IV (mix/total)	1.93 ± 0.44	0.87 ± 0.56	0.000***
WEISS (family)	1.17 ± 0.66	0.41 ± 0.28	0.001**
WEISS (learning and school)	1.10 ± 0.48	0.45 ± 0.28	0.000***
WEISS (life skills)	1.24 ± 0.40	0.59 ± 0.29	0.000***
WEISS (self-management)	1.22 ± 0.88	0.39 ± 0.48	0.008**
WEISS (social activities)	0.91 ± 0.42	0.57 ± 0.34	0.040*
WEISS (adventure activities)	0.46 ± 0.21	0.20 ± 0.16	0.002**

*p < 0.05; **p < 0.01; ***p < 0.001.

### 3.2 DLPFC characteristics of ADHD and neurotypically developed groups

**Figure 3:**
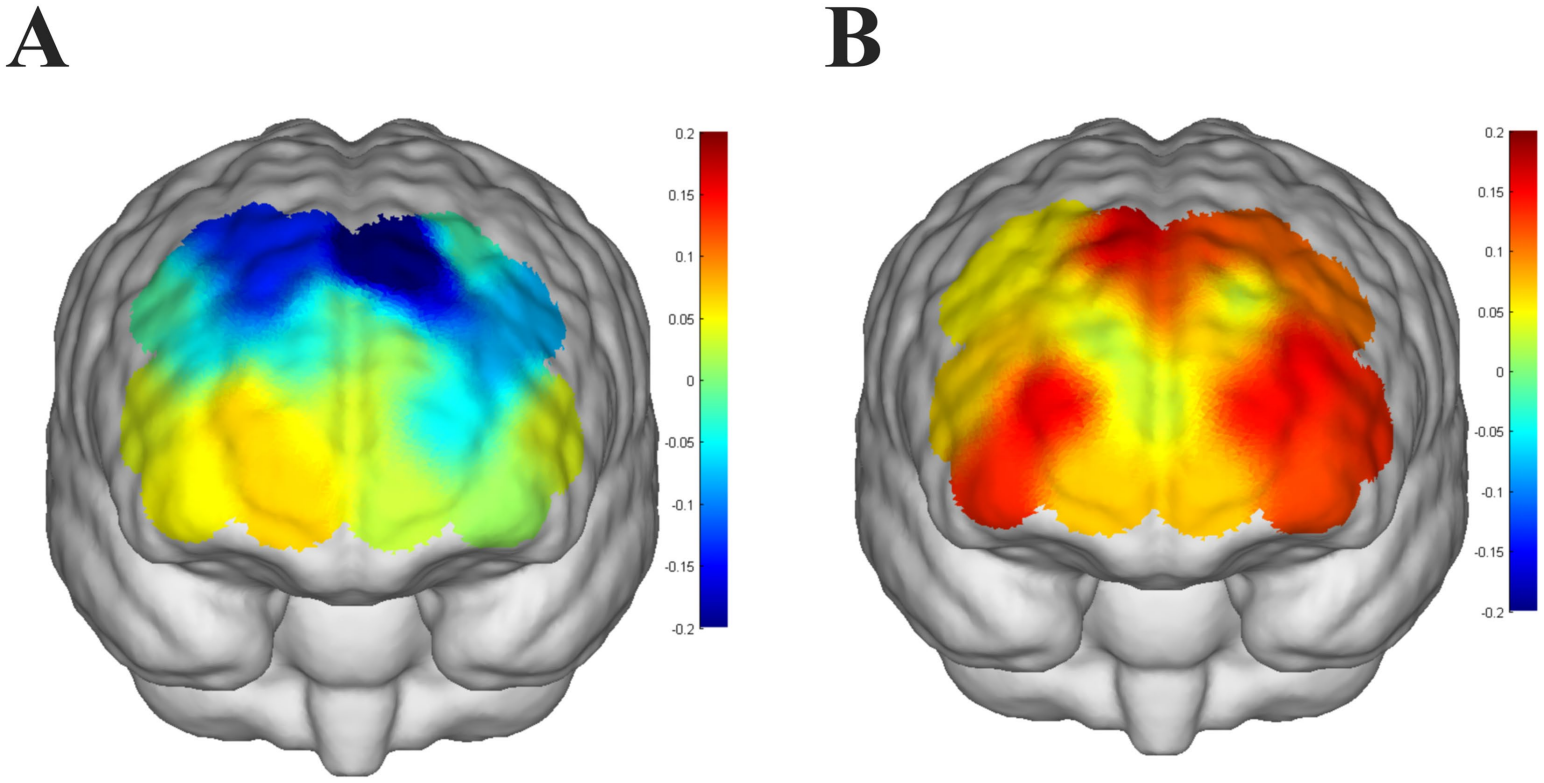
**(A)** Level of brain activation in children with ADHD performing a verbal fluency task, with color bars indicating t-statistics, colors that are approximately cooler indicating lower t-values, and warmer colors indicating higher *t*-values. **(B)** Level of brain activation in neurotypically developed children performing a verbal fluency task, color bars represent t-statistics, colors that are approximately cooler indicate a lower *t*-value, and colors that are warmer indicate a higher *t*-value.

Children with ADHD had lower dorsolateral prefrontal activation and generally showed negative activation bilaterally (Figure 3A), implying that children with ADHD had lower brain activation than at baseline (counting from 1 to 5) when performing the VFT. In contrast, neurotypically developed children had higher dorsolateral prefrontal activation and positive activation bilaterally (Figure 3B), and had higher brain activation than baseline (counting from 1 to 5) when performing the VFT. Children with ADHD had significantly lower activation of channels (CH8 and CH14) in the dorsolateral prefrontal lobes bilaterally compared to neurotypically developed children (*p* < 0.05, FDR corrected).

### 3.3 fNIRS eigenvalues in ADHD and neurotypically developed groups

**Figure 4:**
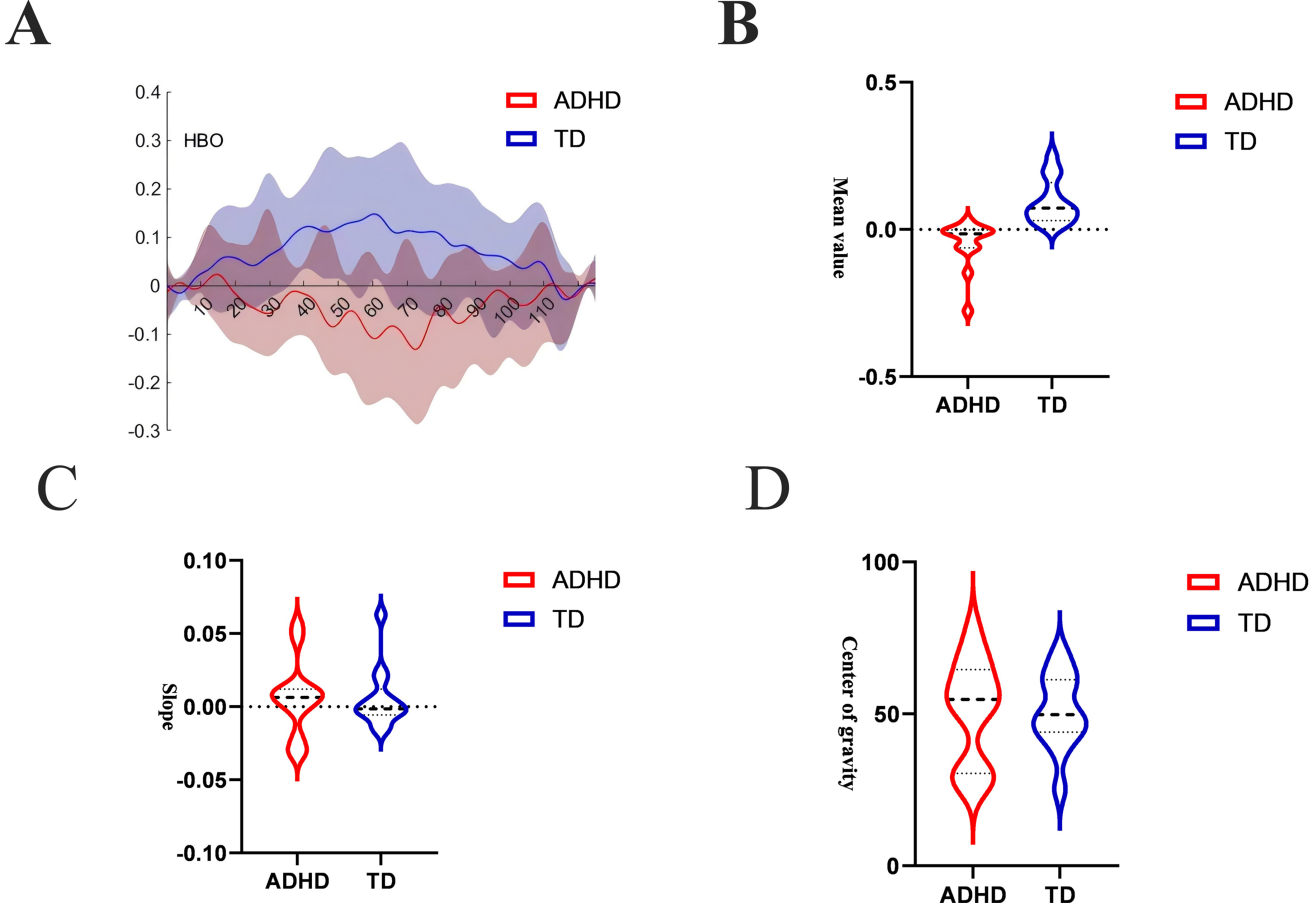
**(A)** HbO time-series curve over the full task sequence. This line plot shows mean HbO concentrations over time in the ADHD group (red) and neurotypically developed group (blue) across a 125-s trial window, including the pre-task baseline (first 30 s), the 60-s active VFT period, and the post-task baseline (final 35 s). Neurotypically developed children displayed a clear task-induced increase in HbO during the activation phase, followed by a gradual return to baseline. In contrast, children with ADHD showed a flat or declining HbO pattern during the task phase. **(B)** Mean HbO amplitude during task period. This bar chart compares the mean HbO signal intensity in the DLPFC during the 60-s VFT activation window between the ADHD and Neurotypically Developed groups. The neurotypically developed group showed significantly higher mean HbO levels compared to the ADHD group (*p* < 0.05). **(C)** Center of gravity (COG) of the HbO signal. This measure captures the temporal distribution of the hemodynamic response, reflecting when the “center” of the activation occurs during the trial period. Both ADHD and neurotypically developed groups showed similar COG values, with peak response occurring in the early-to-mid phases of the VFT period (*p* > 0.05). **(D)** Initial slope of HbO response after task onset. This metric represents the rate of increase (or decrease) in HbO concentration within the first 5 s of the VFT task period. Both groups demonstrated a rapid rise in HbO, and no significant difference was observed in the slope between ADHD and neurotypically developed children (*p* > 0.05).

Figure 4A shows the oxygenation curves of oxyhemoglobin in the ADHD group and the Neurotypically Developed group during the VFTs. The neurotypically developed children showed an upward trend in the oxygenation curves while performing the VFT and a decrease in the oxygenation curves at the end of the task, whereas the children with ADHD showed the opposite trend. The mean values, indicators that reflects the degree of activation during the task, are shown in Figure 4B. Neurotypically developed children had a greater degree of activation, whereas children with ADHD had significantly less activation (*p* < 0.05). COG responded to the temporal bias of the overall level of activation during the performance of the task, and both groups of children showed rapid activation in the first and middle periods of the task; however, there was no significant difference (*p* > 0.05) (Figure 4C). The slope indicator, which responds to the rate of brain activation during the performance of the task, showed that both groups of children had rapid activation during the performance of the task (Figure 4D), but the rate of activation was not significantly different (*p* > 0.05).

### 3.4 fNIRS eigenvalues and behavioral scales in ADHD

**Figure 5:**
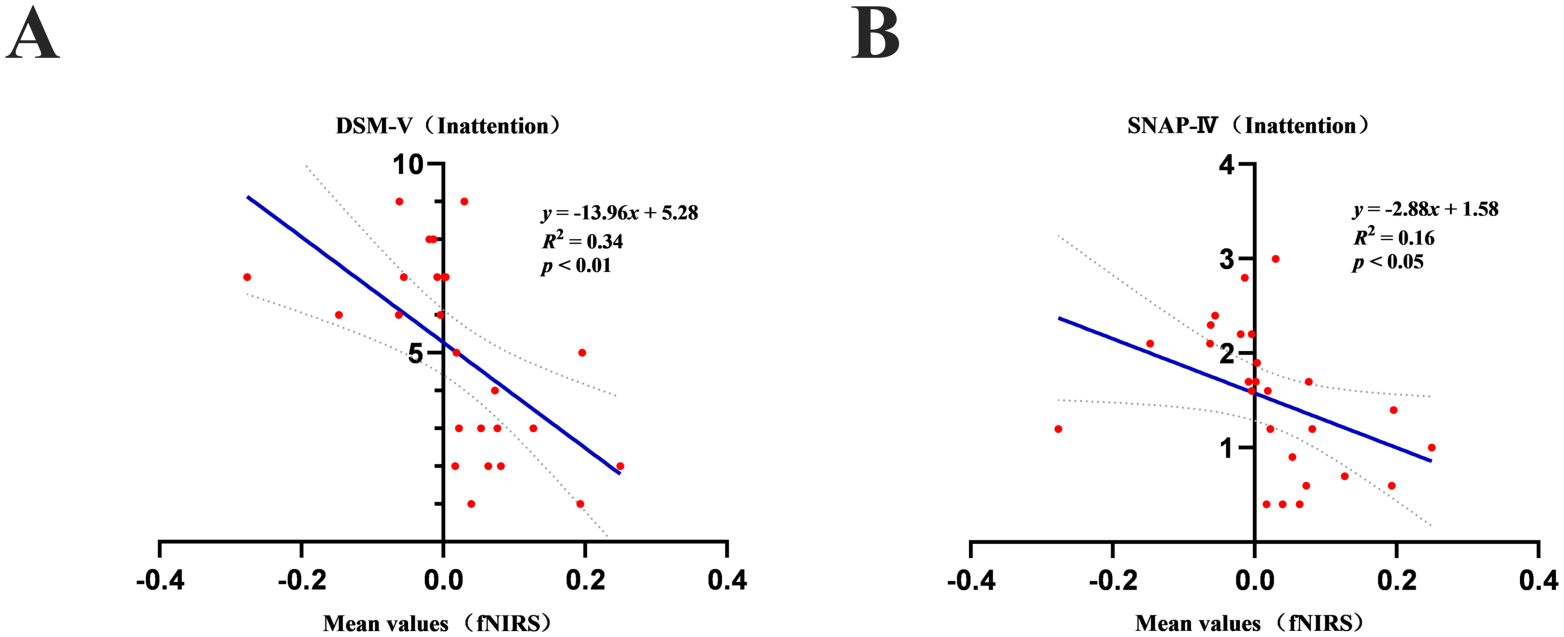
**(A)** Mean values of oxyhemoglobin during a verbal fluency task in children with ADHD and neurotypically developed children correlated significantly (*p* < 0.05) with scores on the DSM-V (Inattention). **(B)** Mean values of oxyhemoglobin during a verbal fluency task in children with ADHD correlated significantly (*p* < 0.05) with scores on the SNAP-IV (Inattention).

By correlation analysis, we found that significant correlations emerged between the mean values of oxygenated hemoglobin and DSM-V (Inattention) scores (Figure 5A) and SNAP-IV (Inattention) scores (Figure 5B) in both groups of children while performing the VFT (*p* < 0.05). However, there were no significant correlations (*p* > 0.05) between the mean values and the other subscales of the DSM-V and SNAP-IV, and between all subscales of the WEISS scale. Furthermore, there were no significant correlations (*p* > 0.05) between the centroid of the mean value of oxyhemoglobin and all subscales of the DSM-V, SNAP-IV, and WEISS-P scale in the children with ADHD and neurotypically developed children during the VFT. The slope values for mean oxyhemoglobin during the VFTs were also not significantly correlated (*p* > 0.05) with all the subscales of the DSM-V, SNAP-IV, and WEISS-P scales.

## 4 Discussion

Our results demonstrate that children with ADHD exhibit significantly lower DLPFC activation during the VFT and that these neural differences are meaningfully associated with behavioral indicators of inattention. This study is the first to explore the use of the VFT paradigm with younger children with ADHD, demonstrating its feasibility and potential to capture task-related differences in cortical activation between children with ADHD and children with neurotypical development. Given that younger children with ADHD face unique challenges in attention and language development, the use of VFT in conjunction with fNIRS provides an innovative approach to assessing these attentional and cognitive domains. This approach fills a gap in existing research and provides a more versatile and developmentally appropriate paradigm for the assessment of younger children. Expanding the scope of the fNIRS paradigm for the study of ADHD may deepen our understanding of the neural mechanisms underlying attention-related symptoms in different age groups.

Children with ADHD exhibited significantly lower activation in the dorsolateral prefrontal cortex (DLPFC) bilaterally, with widespread negative activation during the VFT, whereas neurotypically developed children demonstrated higher activation and positive patterns in the same regions. Both groups showed rapid initial activation and similar temporal activation patterns, as indicated by the COG and slope metrics; however, children with ADHD exhibited significantly reduced mean activation amplitudes. Furthermore, a significant correlation was observed between the mean activation amplitude in the DLPFC and inattention scores on the DSM-V and SNAP-IV scales in the ADHD group.

Children with ADHD exhibited significantly reduced activation and widespread deactivation in the DLPFC during the VFT compared to neurotypically developed children. This aligns with prior research highlighting hypoactivation of the DLPFC in ADHD, a region critical for executive functions such as working memory, cognitive flexibility, and response inhibition (20, 21). The observed widespread deactivation in children with ADHD contrasts sharply with the robust positive activation patterns seen in neurotypically developed children, suggesting fundamental differences in task-related neural engagement. The DLPFC is a core component of the frontoparietal network, implicated in top-down attentional control and goal-directed behavior. Hypoactivation in this region may reflect deficits in maintaining task goals and suppressing irrelevant information, which are hallmark characteristics of ADHD (22, 23). The presence of negative activation, or deactivation, observed in children with ADHD could indicate impaired allocation of cognitive resources or dysfunctional recruitment of auxiliary brain regions to compensate for executive deficits (24, 25). This phenomenon may also reflect disruptions in the DMN, which is known to exhibit aberrant connectivity with the DLPFC in ADHD populations (26). The observed bilateral differences further emphasize the role of interhemispheric functional asymmetry in ADHD. While neurotypically developed children exhibit balanced and coordinated activation across hemispheres during VFTs, children with ADHD demonstrate asymmetrical or deficient engagement, which may exacerbate their difficulties in integrating complex cognitive processes (27). Reduced bilateral DLPFC activation has also been associated with poor performance in VFTs, as this region is critical for semantic retrieval and phonological processing (28). fNIRS demonstrated that children with ADHD exhibit significantly lower levels of activation during mean-response tasks compared to neurotypically developed children. This result aligns with previous studies that have consistently reported reduced cortical activation in ADHD populations during cognitive tasks requiring executive function and attentional control (29, 30). The DLPFC, which is crucial for maintaining task goals, inhibiting irrelevant responses, and integrating information, is particularly implicated in these deficits. Hypoactivation in this region may reflect difficulties in sustaining cognitive effort and engaging task-relevant neural circuits (22, 31). The significant difference in activation levels between ADHD and neurotypically developed children observed in our study may also highlight impaired resource allocation or reduced efficiency in task engagement in the ADHD group. Such findings are consistent with prior fNIRS and fMRI studies showing that children with ADHD struggle to recruit and sustain neural activity in prefrontal regions during tasks that demand consistent focus and self-regulation (23). Moreover, the observed group differences in activation are consistent with theoretical models suggesting that ADHD is characterized by inefficient neural processing and disruptions in the balance between task-positive and default mode networks (32, 33).

Our study indicated no significant group differences in the temporal COG for overall activation during the response task, with both ADHD and neurotypically developed children showing immediate activation in the early-to-mid phase of the task. Additionally, the slope of initial activation, reflecting the speed of post-task brain activation onset, did not differ significantly between the two groups. Both ADHD and neurotypically developed children demonstrated rapid initial activation, suggesting that ADHD-related differences in neural processing may not manifest in the early-phase response dynamics captured by fNIRS. The observed similarity in temporal activation patterns aligns with studies reporting that children with ADHD can engage neural circuits efficiently in task initiation under certain conditions (34, 35). Rapid initial activation may reflect intact low-level sensory and motor processes required for immediate task engagement, which are relatively preserved in ADHD populations (36). Moreover, task-specific demands and the simplicity of initial activation requirements could mitigate the manifestation of executive function deficits during these early task phases (37). However, while the temporal COG and activation slope were comparable, it is important to consider that the quality and sustainability of activation may differ. ADHD-related deficits often emerge during sustained or complex cognitive demands, reflecting difficulties in maintaining engagement and adapting to task demands over time (38, 39). These findings suggest that while early-phase activation may be similar, ADHD-related differences could still arise in other aspects of neural recruitment, such as regional efficiency or interconnectivity (40).

Significant correlations were observed between behavioral measures of inattention and fNIRS-derived cortical activation patterns, with consistent findings across both the DSM-V and SNAP-IV rating scales. This highlights the robust relationship between attentional deficits and neural activation in children with ADHD, particularly during tasks requiring sustained cognitive effort. Our findings align with previous research demonstrating that neuroimaging measures, such as task-related cortical activation, can serve as objective markers of ADHD symptom severity. For instance, studies using fNIRS and fMRI have identified reduced prefrontal activation as a hallmark of inattentive symptoms, reflecting the inability to maintain goal-directed attention and regulate distractors effectively (5, 9). This correlation across multiple behavioral scales underscores the validity of these measures in capturing the attention-deficit characteristic of ADHD, supporting the use of fNIRS as a reliable tool for investigating the neural underpinnings of ADHD symptoms (41–43). The strong associations between inattention scores and neural activation suggest that the attentional deficits measured behaviorally may have a direct neurophysiological basis. This aligns with theoretical models of ADHD, such as the dual-pathway model, which proposes that disruptions in both executive and reward networks contribute to attentional dysfunctions (44–46). The consistency of these findings across different rating scales further strengthens their clinical and diagnostic relevance, as it indicates that both subjective (e.g., SNAP-IV ratings) and diagnostic (e.g., DSM-V criteria) measures capture a shared underlying neurobiological mechanism.

Our results offer significant clinical implications and highlight the potential value of fNIRS in the assessment and management of ADHD. By identifying neural activation patterns associated with VFTs and their correlation with attentional symptoms, this study underscores the utility of fNIRS as a non-invasive and cost-effective tool for evaluating prefrontal cortex activity in clinical populations. Given the challenges associated with diagnosing ADHD, particularly in distinguishing its subtypes and co-occurring conditions, objective neuroimaging markers like those provided by fNIRS could complement traditional behavioral assessments, improving diagnostic accuracy (47–49).

Our results also suggest that fNIRS can provide insights into the neurophysiological mechanisms underlying attention deficits, which could inform targeted interventions. For example, interventions such as cognitive training, behavioral therapy, and pharmacological treatments modulate prefrontal cortex activation, leading to improved attentional control (50–52). By establishing baseline activation patterns and tracking changes over time, fNIRS could serve as a biomarker for treatment efficacy, enabling personalized therapeutic strategies (43, 53, 54). Furthermore, the accessibility of fNIRS makes it particularly valuable in clinical and educational settings where more complex neuroimaging modalities such as fMRI may be impractical. The portability and ease of use of fNIRS systems allow for real-time assessments in naturalistic environments, which is critical for evaluating ADHD-related functional impairments and their impact on daily life (55). Integrating fNIRS with behavioral and clinical assessments could provide a holistic understanding of ADHD, bridging the gap between neurophysiological findings and practical interventions. Future research should expand on these findings by incorporating longitudinal designs to explore how fNIRS markers evolve with development and intervention. Combining fNIRS with advanced analytical methods, such as connectivity analysis and machine learning, could further enhance its diagnostic and predictive capabilities (56). Additionally, integrating fNIRS with multimodal approaches, including EEG and behavioral metrics, may provide a comprehensive understanding of ADHD’s neural and behavioral dimensions (14, 57). Such advancements could pave the way for more precise and effective diagnostic and treatment frameworks, ultimately improving outcomes for children with ADHD.

This study highlights the potential of using fNIRS to study the VFT paradigm in younger children with ADHD. fNIRS is increasingly being used as an adjunctive diagnostic tool in ADHD research, not least because of its ability to non-invasively measure prefrontal cortical activity during cognitive tasks (58). However, a limited task paradigm for younger children with ADHD is the Go/NoGo paradigm, with the Go/NoGo task being the most commonly used framework for assessing inhibitory control and attentional regulation (59). In contrast, the VFT paradigm has been used primarily with adolescents and adults with ADHD to assess emotion regulation and more complex cognitive functions (60).

Beyond its research utility, fNIRS holds considerable promise for clinical application in the diagnosis and management of ADHD. Its ability to objectively quantify cortical activation provides a valuable complement to traditional behavioral assessments, particularly in differentiating ADHD subtypes that often present with overlapping symptoms (61, 62). Moreover, the portability, affordability, and tolerance to motion artifacts make fNIRS especially suited for use in real-world environments, such as schools or outpatient clinics (63–65). This enhances accessibility for broader populations and supports the integration of neurophysiological markers into routine clinical practice, potentially enabling earlier identification and personalized intervention strategies for children with ADHD. Taken together, these insights reinforce the value of fNIRS as a neuroimaging tool for studying executive dysfunction in ADHD and suggest new avenues for objective assessment. Future studies should further validate the use of the VFT paradigm as a diagnostic tool in larger samples of younger children with ADHD and explore its application in other neurodevelopmental settings. This will pave the way for more comprehensive and targeted assessments and the development of better ADHD-assisted diagnostic models.

This study is not without limitations. One notable limitation is the relatively small sample size, which may affect the robustness and generalizability of the findings. This stems from the pilot nature of the study, which was designed to assess the feasibility of applying the VFT in combination with fNIRS in younger children with ADHD, as well as to preliminarily explore associated neural activation patterns. Due to its exploratory scope, the study prioritized methodological validation over statistical power for broad generalization. Additionally, data collection took place between January and September 2022, a period during which Shanghai faced strict public health restrictions due to the COVID-19 pandemic. This significantly constrained our ability to recruit a larger cohort. Several influential fNIRS studies in ADHD populations have employed similarly limited sample sizes during their preliminary validation stages, particularly in pediatric contexts (66–71). Nonetheless, small sample sizes inherently increase the risk of type I and type II errors and may reduce the stability of effect estimates. Therefore, the results of this study should be interpreted with caution. Another drawback of fNIRS that is not unique to this study is that it measures signals approximately 1.5 cm deep into the cortex. This means that the contribution of subcortical structures to the performance of speech fluency tasks by children with ADHD as well as by neurotypically developed children could not be captured in this study. Additionally, our probe did not include short-separated channels, which meant that we could not regress physiological noise from hemodynamic responses. Another limitation is our use of a DPF of 6 for all participants. Although this convention facilitates comparison across studies, it neglects inter-individual and region-specific variability in photon propagation, especially in a pediatric sample where head size, skull thickness, and tissue optical properties change markedly with age. By applying a single DPF value, we may have introduced systematic bias into our estimates of hemoglobin concentration changes, potentially under- or over-estimating true activation amplitudes in some channels. Further studies are needed to assess how these methodological choices generalize across broader developmental stages and clinical populations. Despite these challenges, the findings in this study pave the way for future investigations of functional brain imaging in children with ADHD by fNIRS, using high-density probes and larger sample sizes, as well as more complex and diverse cognitive and language tasks.

## 5 Conclusion

We investigated the neural correlations between VFT in children with ADHD using fNIRS. Children with ADHD had significantly lower activation in DLPFC compared to neurotypically developed children, and this was accompanied by a broad pattern of negative activation during the task. Mean activation amplitude showed significant correlations with behavioral measures of inattention on the DSM-V and SNAP-IV scales, highlighting the close relationship between cortical activation deficits and attention deficits. These findings highlight the potential of fNIRS as a non-invasive, cost-effective tool for assessing attention-related neurological deficits in younger children with ADHD and may help to establish a diagnostic tool for children with ADHD. By combining neurophysiological measures with behavioral assessments, fNIRS offers a promising avenue to improve diagnostic accuracy and tailoring interventions. Future research should build on our findings by investigating longitudinal changes in neural activation, expanding functional assessment tools for ADHD, and extending the paradigm for the use of fNIRS as a diagnostic tool for children with ADHD, particularly for core dysfunctions such as executive function and attention.

## Data Availability

The raw data supporting the conclusions of this article will be made available by the authors, without undue reservation.
